# Microstructural Evolution, Tensile Failure, Fatigue Behavior and Wear Properties of Al_2_O_3_ Reinforced Al2014 Alloy T6 Heat Treated Metal Composites

**DOI:** 10.3390/ma15124244

**Published:** 2022-06-15

**Authors:** V. Bharath, V. Auradi, G. B. Veeresh Kumar, Madeva Nagaral, Murthy Chavali, Mahmoud Helal, Rokayya Sami, NI Aljuraide, Jong Wan Hu, Ahmed M. Galal

**Affiliations:** 1 Department of Mechanical Engineering, Sri Venkateshwara College of Engineering, Bengaluru 562157, Karnataka, India; bharathv88@gmail.com; 2 Siddaganga Institute of Technology, Visvesvaraya Technological University, Tumakuru 572104, Karnataka, India; vsauradi@gmail.com; 3 National Institute of Technology—Andhra Pradesh, West Godavari (Dist.), Tadepalligudem 534101, Andhra Pradesh, India; 4 Aircraft Research and Design Centre (ARDC), HAL Vimanapura Post, Marathahalli, Bangalore 560037, Karnataka, India; madev.nagaral@gmail.com; 5 Office of the Dean (Research) & Division of Chemistry, Department of Science, Faculty of Science & Technology, Alliance University (Central Campus), Chandapura-Anekal Main Road, Bengaluru 562106, Karnataka, India; 6 Department of Mechanical Engineering, Faculty of Engineering, Taif University, P.O. Box 11099, Taif 21944, Saudi Arabia; mo.helal@tu.edu.sa; 7 Production Engineering and Mechanical Design Department, Faculty of Engineering, Mansoura University, Mansoura P.O. Box 35516, Egypt; 8 Department of Food Science and Nutrition, College of Sciences, Taif University, P.O. Box 11099, Taif 21944, Saudi Arabia; rokayya.d@tu.edu.sa; 9 Department of Physics, Turabah Branch, Turabah University College, Taif University, Taif 21944, Saudi Arabia; n.aljareed@tu.edu.sa; 10 Department of Civil and Environmental Engineering, Incheon National University, Incheon 22012, Korea; 11 Incheon Disaster Prevention Research Center, Incheon National University, Incheon 22012, Korea; 12 Mechanical Engineering Department, College of Engineering, Prince Sattam Bin Abdulaziz University, Wadiaddawaser 11991, Saudi Arabia; ahm.mohamed@psau.edu.sa

**Keywords:** Al2014, Al_2_O_3_, heat treatment, mechanical properties, fatigue studies

## Abstract

The paper focused on an experimental study on the microstructural, mechanical, and wear characteristics of 15 wt.% alumina (Al_2_O_3_) particulates with an average particle size of 20 µm, reinforced in Al2014 alloy matrix composite as-cast and heat-treated samples. The metal matrix composite (MMC)samples were produced via a novel two-stage stir-casting technique. The fabricated composite samples were subjected to evaluate hardness, tensile strength, fatigue behavior and wear properties for both as cast and T6 heat-treated test samples. The Al2014 alloy and Al2014-15 wt.% Al_2_O_3_ MMCs were in solution for 1 h at a temperature of 525 °C, quenched instantly in cold water, and then artificially aged for 10 h at a temperature of 175 °C. SEM and X-ray diffraction analyses were used to investigate the microstructure and dispersion of the reinforced Al_2_O_3_ particles in the composite and the base alloy Al2014. The obtained results indicated that the hardness, tensile and fatigue strength and wear resistance increased when an amount of Al_2_O_3_ particles was added, compared to the as-cast Al2014 alloy and it was observed that after subjecting the same composite samples to heat treatment, there was further enhancement in the mechanical and wear properties in the Al2014 matrix alloy and Al2014-15 wt.% Al_2_O_3_ composite samples.

## 1. Introduction

Aluminum (Al) alloys are nonferrous materials that are used in engineering sectors because of their desirable properties, such as high ductility, good resistance to corrosion, decent strength to weight ratio, and relatively lower cost [1]. Al alloys are categorized as wrought and cast alloys and furthermore, they are grouped into heat treatable and un-heat treatable alloys [2]. Al wrought products are exposed to plastic deformation by the process of the hot and cold working process. The Al2014 alloy is the one type of wrought alloy with copper (Cu) as the major alloying element. Due to the presence of Cu, it reduces the ductility and corrosion resistance, enhances the strength, and promotes precipitation hardening [3]. In the current study, the Al2014 alloy is opted for because of its good strength, higher mechanical properties, practical usages, ability to cast, etc. Hence, Al2014 alloys are used in aerospace, military vehicles, and rocket fins [4,5]. Nevertheless, their uses were always limited, as traditional Al alloys are soft and well known for their lower wear resistance. This issue can be resolved by the addition of hard ceramic strengthening, and reinforcing particulates in Al alloys to create discontinuous reinforced MMC with almost isotropic characteristics. Numerous researchers have focused on Al MMCs reinforced with ceramic particulates in recent years. Due to the low density and melting point, higher specific strength, and thermal conductivity of Al alloys, a wide range of hard ceramics, such as silicon carbide (SiC) [6,7], boron carbide (B_4_C) [8], Al_2_O_3_ [9], titanium carbide (TiC) [10] and graphite (Gr) [11], in various forms, such as whiskers, particulates, or fibers [12], have been reinforced into the alloys. They may be modified to have better characteristics, such as higher specific strength and rigidity, enhanced resistance to wear, stronger thermal and mechanical fatigue, and resistance to creep than those of alloys with improved high-temperature performance. Researchers have reported problems related to MMCs leading to a wide scatter in the ultimate strength and ductility, due to the non-consistency of reinforcing particulate distribution [13,14,15]. Micro-meter-sized ceramic particulates have been effectively used to manufacture Al MMCs through different techniques, such as powder metallurgy [16], liquid metal infiltration [17], and squeeze casting [18]. Out of the above process, fabricating Al MMCs with a discontinuous reinforcement stir casting process is the most suitable due to its decent bonding between matrix and reinforcing particles, simpler matrix structure regulations, ease of fabrication and cost-effective, closer net shape and is suitable for mass production compared to all other casting methods [19]. Generally, stir casting represents the addition of ceramic particulates into a molten matrix in a single step [20,21]. Bharath et al. studied the Al2014-Al_2_O_3_ composites made via stir casting and the results presented that the micro-hardness of the Al2014 alloy improved after adding Al_2_O_3_ particles and microhardness enhanced with an increase in the addition of reinforcement [22]. Guo et al. [23] stated that a very important aspect in determining the tensile strength of Al alloys and short Al_2_O_3_ fiber composites is the interfacial bond. The tensile behavior of Al-Al_2_O_3_/B_4_C composites formed by the technique of infiltration was stated by Kouzele et al. [24]. Ma et al. [25] stated that for hot extruded Al_2_O_3_, TiB_2_/Al-Cu composite, 2.2% of elongation is achieved. Chai-Chaw Perng et al. [26] stated low cycle fatigue activity of Al6061-Al_2_O_3_ hot expelled composites under T-6 pressure. The fatigue strength of the Al6061/Al_2_O_3_ T-6 composite is stated to be in feriorto that of the unreinforced Al6061-T6 alloy, particularly in the region of high amplitude and short life. Hochreiter et al. [27] examined the fatigue behavior of Al6061 extruded alloy, Al6061-10 wt.% of SiC extruded, and Al6061-10 wt.% Al_2_O_3_ composites. It was observed that in the low and high-cycle fatigue region, Al6061-SiC MMC’s fatigue life was higher compared to the Al6061-Al_2_O_3_ composite. Senthilkumar et al. [28] found that the failure period was higher for the composites reinforced by nano Al_2_O_3_ compared to micro-sized Al_2_O_3_ MMCs, due to the lower order plastic strain induced. Hoskins et al. [29] experimented on Al2014 and Al2024 alloys strengthened with Al_2_O_3_ and SiC and reported that adhesive wear rate declines with an increase in the particle content (for a given particle size). SiC is more effective in wear resistance as contrasted to Al_2_O_3_. Wear resistance improved with improved SiC content within the matrix alloy for a constant SiC particle size. Heat treatment has good features for homogenizing and refining eutectic microstructures and increasing alloy characteristics at a reasonable cost and with ease of use. A considerable amount of dimples were observed throughout the fractured surface after T6 heat treatment, indicating a highly ductile fracture. The alloy shows an improved hardening response due to the refining of the eutectic structure and precipitation of nanoparticles in the Al matrix [30]. Maxim et al. [31] have found that homogenizing and artificial aging of T6 and T7 improves the Al composite strength by 20% relative to natural aging. They stated that fine ceramic particles lead to a strong connection with tensile resistance and heat treatment. Elmas [32] examined the aging performance of a spray cast Al7075 alloy. The solution treatment was conducted at 470 °C for 30 min, followed by water quenching and later aged at different temperatures for a chosen time. They noticed that aging increases with the increments in temperature and they stated that good properties are achieved at low temperatures. Daud et al. [33] conducted experiments on Al7075 reinforced with Al_2_O_3_ particles using the liquid metallurgy technique and the samples were subjected to heat treatment at a particular temperature for 2 h for solution treatment and then subjected to water quenching, also tempered at 120 °C for 8 h. They concluded that there was an enhancement in the hardness, strength, and good resistance to wear as compared to a matrix alloy. However, the literature reports that thermal treatment has a higher impact on the microstructure and mechanical characteristics of casted Al MMCs, and the process variables rely on the chemical composition. Therefore, limited data are accessible to assess the result of heat treatment on characteristics of Al MMCs on the mechanical and wear characteristics of Al2014 alloy reinforced with Al_2_O_3_ MMCs, processed by an innovative two-stage liquid stirring process. With an increase in the requirement of advanced materials in advanced modern applications, Al-based MMCs are considered a contender as are placement for ferrous and nonferrous materials. Classic examples are cylinder liners of vehicle motors and brake rotors. Al2014 alloy reinforced with Al_2_O_3_ composites is predominantly utilized in the aircraft and automobile sectors and more specifically, it is used in aerospace applications in the construction of structural frames (i.e., wing to fuselage attachment root fittings and bulkhead), which has increased with the advent of advanced liquid stirring processes. However, there is a need to develop a new formulation and evaluation of its properties. In the current study, Al2014-Al_2_O_3_ composites are produced by a novel two-stage stir casting in which ceramic particles were added into a molten alloy in two stages, instead of incorporating them at a specific time. This novel two-stage mixing enhances the wettability of the reinforced Al_2_O_3_ particles in the matrix material [34]. Improving wettability and good casting leads to improvements in the properties of Al MMCs.

Furthermore, the MMCs produced are subjected to heat treatment along with the as-cast Al2014 alloy to understand the further enhancements in the mechanical, fatigue, and wear properties.

## 2. Preparation of the Al2014-Al_2_O_3_ MMCs and Experimentation Details

### 2.1. Details of Matrix and Microparticulate Reinforcement Materials

The matrix for the current studies selected was Al2014, which belongs to the Al2xxx series (which is the Al and Copper (Cu) series) and ingots of the same series were acquired from Fenfee Metallurgicals, Bangalore, India. Table 1 demonstrates the chemical composition of the obtained alloy Al2014. The reinforcement materials selected were fine particulates of Al_2_O_3_ of size 20 µm, provided by Fenfee Metallurgicals, Bangalore, India, and Table 2 demonstrates the characteristics of the matrix and reinforcement materials considered for the current investigative studies.

### 2.2. Preparation of the Al2014-Al_2_O_3_ MMCs

In the present investigative studies, the Al2014 reinforced with 15 wt.% Al_2_O_3_ MMCs were prepared using the most economical and widely used liquid metallurgy method by the stir casting technique, in which a novel two-stage stir casting technique was adopted. Initially, a weighed amount of Al2014 alloy was taken in a Gr crucible, placed in an electrical resistance furnace, and heated to 725 °C temperature. Once this temperature was accomplished, hexachloroethane (C_2_Cl_6_) degassing tablets were added to avoid the entrapment of the gas while stirring and a 15 wt.% weighted quantity of preheated Al_2_O_3_ was introduced into the melt in two stages, rather than adding it into the melt at once to avoid the agglomeration and improve the distribution of Al_2_O_3_ particles throughout the alloy matrix. Vigorous stirring was carried out using a zirconia-coated steel impeller, which was maintained at 250 rpm at 10 min. Later, the melt was poured into the prepared mold of size 125 mm in length and 12.5 mm in diameter and allowed for solidification. After solidification, Al2014-15 wt.% Al_2_O_3_ cylindrical-shaped MMCs were obtained.

### 2.3. Experimental Details

The obtained Al2014-15 wt.% Al_2_O_3_ MMCs were subjected to machining so as obtain the test samples as per ASTM standards. The micro-hardness test was conducted as per ASTM E10 on the finely polished cast and Al2014-15 wt.% Al_2_O_3_ MMCs were tested using the Vickers hardness tester of Zwick/RoellIndtech (ZHVµ) of Germany. The tests were conducted with a load of 300 g (HV 0.3) spread over the 15 mm diameter and 10 mm length specimen for a dwelling period of 10 s. The study was performed at ten different locations to confirm the possible impact of indenter lying on tougher particles. The average of all ten measurements was taken as the hardness of the sample. The ultimate tensile strength tests were performed by ASTM E8 using an INSTRON-5980 model of a USA-made computerized universal testing machine (UTM), 60 KN capabilities with a least count of 4 N. All the tests were performed in a displacement mode at a rate of 0.1 mm/min. Three experiments were carried out, and the average value was reported. Some of the matrix tensile behaviors and their composites, viz, UTS, YS, and ductility were assessed. For the microstructural studies, the fracture surfaces were presented after the study, using SEM to understand the mechanism of fracture. Wear tests were performed to assess the materials’ wear behavior.

The dry sliding wear tests were performed on Al2014 alloy, Al2014-Al_2_O_3_ reinforced MMCs by the standard ASTM G99 [35,36] using a computerized pin-on-disk tribometer of DUCOM Instruments Pvt. Ltd., Bangalore India (model: TR20LE). The wear testing device was equipped with an EN-32 steel counter disk, with HRC65 hardness and 160 mm maximum track diameter. The cylindrical samples with a diameter of 8 mm and a height of 25 mm were used for the tests. The wear behavior of the composites under various conditions was considered in the present study. Wear testing was carried out using parameters such as load (N), sliding speed (rpm), and sliding distance (m). To understand the wear mechanism, worn surfaces and debris of both as-cast and heat-treated test samples were subjected to optical studies to understand the dominant wear mechanism. The fatigue tests were conducted on BISS, Bangalore India MTL environment with 2350 controller. The fatigue behavior of the MMCs was tested using a low-cycle fatigue testing machine; the fatigue tests were conducted as per ASTM-E606 at both room and heat-treated conditions, with a constant frequency of 50 Hz and a tension ratio (R) of −1. Fatigue life (N_f_) is considered when the number of cycles leads to separation of the samples or complete failure. On a gauge portion of the test samples, a constant surface finish of 5 μm was retained to minimize the surface finishing impact and irregularities by increasing the fine grits of the emery paper for all the produced samples. The cumulative stresses applied throughout the tests ranged between 50 and 200 MPa, corresponding to 50–90% of the material strength [37]. For better results, average values of the three readings were taken for the Al2014 alloy and Al2014-15 wt.% Al_2_O_3_ MMCs at both as-cast and heat-treated samples, respectively. Heat-treated Al2014-15 wt.% Al_2_O_3_ MMCs were subjected to the T6 condition, which involves a solutionizing process at a temperature of 525 °C for 1 h. Immediately, it is followed by water quenching at room temperature and later is pre-aged to room temperature for 2 h finally, the composite samples are subjected to artificial ageing at 175 °C for 10 h in a muffle furnace. The microstructural analysis of the composites was carried out using a TESCAN VEGA 3 LMU, Czech Republic scanning electron microscope (SEM). For SEM/EDX examination, the machine was connected to the JED 2300 software programmer to identify the distribution of the particles, accompanied by energy dispersive spectroscopy (EDS). The X-ray diffractometer (XRD) analysis was performed by PANALYTICAL XRD (Cu-Ka radiation at a scan speed of 0.011 m/s). The 2θ range is designed to cover all the intense peaks of the material phases that are predictable. The SEM images of the worn surfaces and the fractured specimens were taken to study the type of wear occurring and the type of fracture.

## 3. Results and Discussions

### 3.1. Microstructural Studies

**Figure 1:**
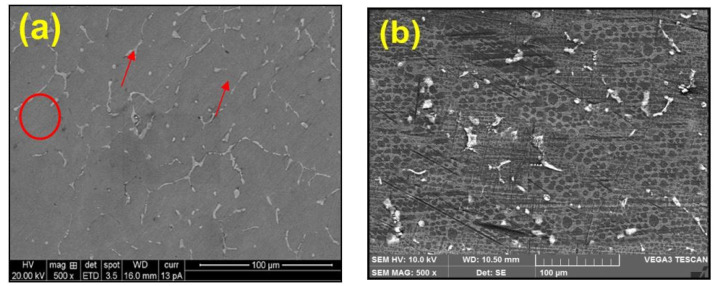

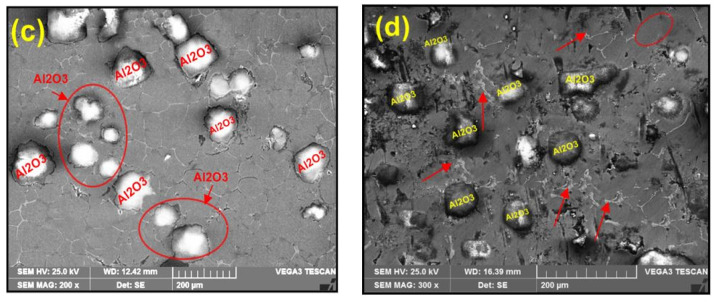
(**a**–**d**) SEM microphotographs of (**a**) as-cast Al2014 alloy (**b**) as-cast T6 heat-treated Al2014 alloy (**c**) as-cast Al2014-15 wt.% Al_2_O_3_ and (**d**) Al2014-15 wt.% Al_2_O_3_ both at room and T6 heat-treated, respectively.

Figure 1a,b depict the microstructure of the as-cast Al2014 alloy, and Figure 1c,d show the Al2014-15 wt.% Al_2_O_3_ particles with an average particle size of 20 µm before and after heat treatment. Figure 1a shows an un-heat treated Al2014 alloy SEM micrograph, reflecting Al dendrites and precipitate in interdendritic regions along the boundaries of the Al dendrite (marked by a circle symbol) and the precipitates in the interdendritic regions (marked by arrows), whereas Figure 1b shows the heat-treated Al2014 alloy and depicts the Al phase black in color and the precipitate is white-colored, dispersed in the Al matrix.

Figure 1c,d shows the uniform dissemination of the Al_2_O_3_ reinforced particles in the produced Al2014-15 wt.% Al_2_O_3_ composites before and after heat treatment. Secondary intermetallic phases and reinforced Al_2_O_3_ particles can be easily identified in the matrix of the T6 heat treatment, as shown in Figure 1d, i.e., the light grey areas (marked by a circle) are primary α-Mg or α-Cu or α-Si phases, whereas the dark grey areas (shown by arrows) are secondary intermetallic precipitates of Al_2_Cu or Al_2_Mg or Mg_2_Si phases and the extent of uniform dissemination of the Al_2_O_3_ particles in heat-treated composites. Figure 1d is more uniform when contrasted to the composite without heat treatment (Figure 1c), suggesting less agglomeration, due to the minimizing porosity level and good relationship with the matrix and the reinforcing Al_2_O_3_ particles, even after heat treatment. The shape of the Al_2_O_3_ particles in unheated-treated composites (Figure 1c) is irregular but after the T6 treatment, the shape of the Al_2_O_3_ particles is spherical, as it is shown in Figure 1d. In addition to reinforcing the particles’ ageing kinetics, phase formation and precipitation during the heat-treatment process increase alloy strengthening [38,39,40,41,42,43].

**Figure 2:**
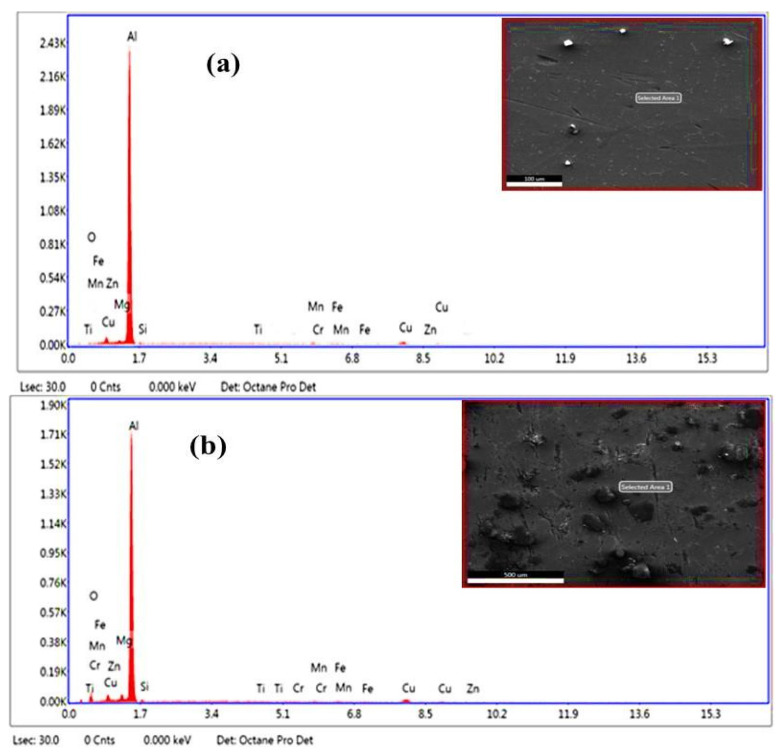
(**a**,**b**). EDS spectrum of (**a**) Al2014 after T6 treatment; (**b**) Al2014-15 wt.% Al_2_O_3_ MMCs after T6 treatment.

The chemical composition of the heat-treated as-cast Al2014 alloy and 15 wt.%of Al_2_O_3_ are shown in Table 3. Figure 2a shows the EDS spectrum of heat-treated Al2014 alloy at 525 °C and aged at 175 °C. Traces of Cu, Mg, and Si are found as primary alloying elements on the Al interfaces and are confirmed by the EDS spectrum. Cu and Mg are usually applied to the Al alloy for age-hardening by the precipitation of Al_2_Cu, Al_2_Mg, and Mg_2_Si precipitates. Figure 2a shows Al-Cu-Mg-Si precipitates because of heat treatment, along with the Mg, Cu, and Si peaks. Figure 2b shows the elemental analysis of heat-treated Al2014-15 wt.% of the Al_2_O_3_ composite, which confirms the elements such as O, Mg, Si, Ti, Mn, Fe, Cu, Cr, and Zn. Dispersion of Al_2_O_3_ (Figure 2b) in Al2014 is confirmed by the existence of oxygen peaks.

**Figure 3:**
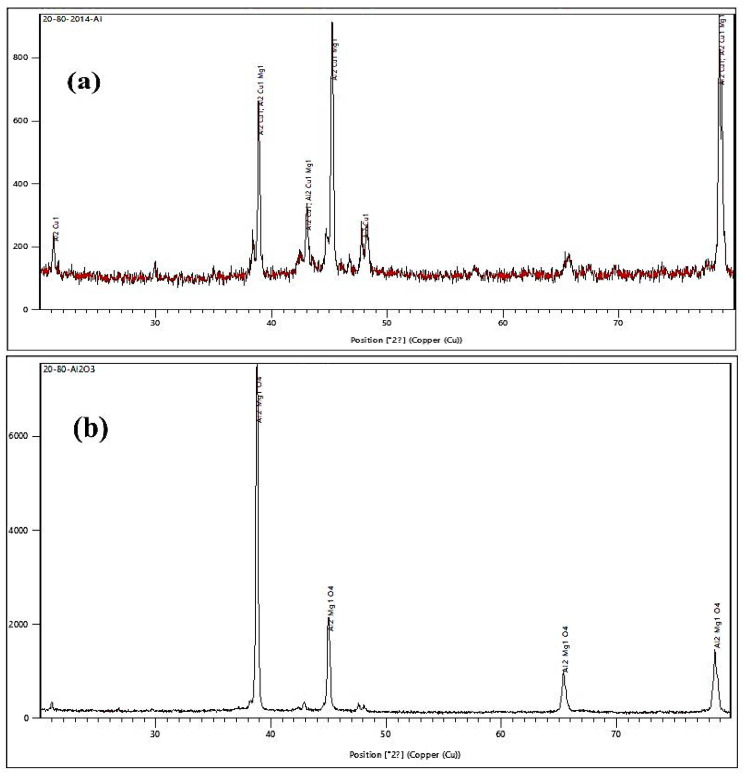
XRD pattern of (**a**) Al2014 after T6 heat treatment; (**b**) Al2014-15 wt.% Al_2_O_3_ MMCs T6 heat treatment.

Figure 3a,b depicts the XRD pattern of the heat-treated Al2014 alloy Al2014 alloy reinforced with 15 wt.% of Al_2_O_3_ composite to detect the Al_2_O_3_ presence and other intermetallic phase formation. From Figure 3a,it can be observed that the peaks at 21.1816° and 47.9172° belong to Al_2_Cu_1_ (reference code: 98-018-6680) and the other peaks at 38.8°, 43.0°, 45.2°, and 78.6° belong to Al_2_Cu_1_, Al_2_Cu_1_Mg_1_ (reference code: 98-005-7693) and the other remaining small peaks are ascribed to impurity. From Figure 3b, it can be observed that the peaks 38.7°, 42.8°, 44.9°, 65.4°, and 78.5° belong to Al_2_Mg_1_O_4_ (reference code: 98-009-6833), and other remaining minor peaks are ascribed to impurity.

### 3.2. Hardness Measurements

**Figure 4:**
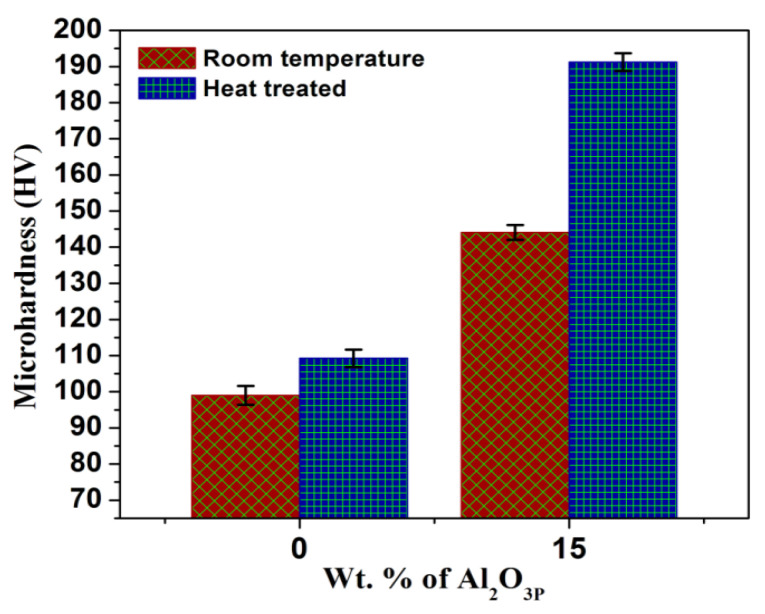
Comparisons of microhardness values of Al2014 and Al2014-15 wt.% of Al_2_O_3_ composite with a fine particle, before and after heat treatment condition.

The microhardness of the Al2014 alloy and Al2014-15 wt.% Al_2_O_3p_ composites before and after heat treatment are depicted in Figure 4 and their corresponding values of standard deviation. The heat treatment has significant effects on the microhardness of Al2014 alloy and Al2014-15 wt.% Al_2_O_3_ reinforced MMCs. A solutionizing temperature of 525 °C with a duration of about 1 h and an aging temperature of 175 °C with a duration of 10 h significantly alters the microhardness of both the Al2014 matrix alloy and Al2014-15 wt.% Al_2_O_3_ filled MMCs. Figure 4 shows the enhancement in microhardness of the heat-treated Al2014 alloy (109.26 ± 2.42), as contrasted with the Al2014 alloy (99 ± 2.63) before heat treatment. This is due to the substantial improvements in Cu solubility in Al due to the solution temperature treatment. The Al-rich phase will also contain Cu at room temperature in a supersaturated solid solution. Fine Al_2_Cu particles formed and precipitated into solution during aging. Therefore, due to the artificial age hardening, the hardness of the alloy improved [44]. In addition, Figure 4 shows the microhardness of both un-heat treated and heat-treated Al2014-15 wt.% Al_2_O_3_ MMCs (144.10 ± 2.06 and 191.24 ± 2.45), which is increased after the addition of Al_2_O_3_ particles. The enhancement in microhardness is primarily because of the increment in the intensity, hindering the motion of the dislocation by the hard ceramic Al_2_O_3_ particles. In addition, the microhardness of the composite with the T6 treatment condition is substantially higher than that of the composite without heat treatment. This happens because, during the aging process, the development of secondary phase precipitates will effectively obstruct the motion.

It is also observed that heat treatment has a considerable impact on the improvement in the microhardness of both the as-cast Al2014 alloy and Al2014-15 wt.% Al_2_O_3_ composites. An improvement of about 10.36% and 24.64% has been observed for the heat-treated Al2014 alloy and Al2014-15 wt.% Al_2_O_3_ MMCs, when compared with the untreated Al2014 alloy and Al2014-15 wt.% Al_2_O_3_ MMCs, respectively. This improvement is due to the presence of maximum Cu, Mg, and Si contents, which form intermetallic compounds with ceramic particles. Similar trends were observed by many other researchers [31,45]. The improvement in the microhardness of the matrix alloy and MMCs is also because some of the present minor casting defects are cured by improving the microhardness of the composite samples treated with the T6 heat-treatment procedure.

### 3.3. Ultimate Tensile Strength (UTS), Yield Strength (YS), and Percentage Elongation

**Figure 5:**
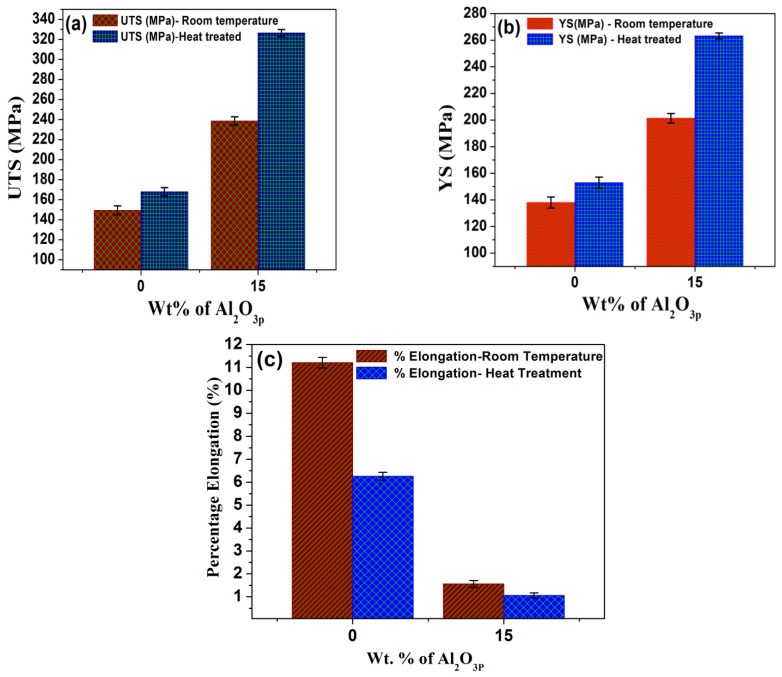
(**a**,**b**) Comparisons of UTS and YS values of Al2014 and Al2014-15 wt.% of Al_2_O_3_ composites before and after heat treatment. (**c**) Comparisons of percentage elongation values of Al2014 and Al2014-15 wt.% Al_2_O_3_ composites both at as-cast and heat-treated conditions.

The comparison of UTS, YS, and percentage elongation of the base alloy (Al2014) and Al2014 reinforced with 15 wt.% Al_2_O_3_ particles before and after the heat-treatment condition is shown in Figure 5a–c.

The T6 heat treatment has significant effects on the UTS and YS of Al2014 and Al2014-15 wt.% Al_2_O_3_ composites. A solutionizing temperature of 525 **°**C with a duration of about 1 h and an aging temperature of 175 °C with a duration of 10 h significantly alters the UTS and YS of both Al2014 matrices alloys and Al2014-15 wt.% Al_2_O_3_ MMCs. Figure 5a shows the enhancement in the UTS of the heat-treated base alloy (i.e., 167.90 ± 4.16 MPa) and Al2014-15 wt.% Al_2_O_3_ MMCs (i.e., 326.39 ± 3.56 MPa) as contrasted to the base alloy (149.29 ± 4.53 MPa) and Al2014-15 wt.% Al_2_O_3_ composite (238.54 ± 4.18 MPa) before heat treatment, respectively. An improvement in UTS of about 12.46% and 36.82% is noticed in the heat-treated base alloy and Al2014-15 wt.% Al_2_O_3_ composite, respectively, as contrasted to the base alloy, the Al2014-15 wt.% Al_2_O_3_ MMCs without heat treatment. Figure 5b shows the enhancement in YS of the heat-treated base alloy (152.89 ± 4.28 MPa) and Al2014-15 wt.% Al_2_O_3_ MMCs (263.23 ± 2.23 MPa) as contrasted to the base alloy (138.01 ± 4.19 MPa) and Al2014-15 wt.% Al_2_O_3_ composite (201.29 ± 3.56 MPa) before heat treatment, respectively. An improvement in the YS of about 10.78% and 30.77% in the heat-treated base alloy and Al2014-15 wt.% Al_2_O_3_ composite is noticed, respectively, as contrasted to the base alloy and Al2014-15 wt.% Al_2_O_3_ composite without heat treatment. This enhancement in UTS and YS of the heat-treated Al2014 alloy and Al2014-15 wt.% Al_2_O_3_ MMCs can be attributed to their increased hardness, as discussed in the previous Section 3.2. The enhancement in UTS and YS is also induced by the difference in the thermal expansion coefficient between the Al2014 alloy matrix and the Al_2_O_3_ particle, which can lead to maximum dislocation density and the fine Al_2_Cu can be precipitated after T6 treatment to enhance the UTS.

To disperse the alloying elements and increase the uniformity of the microstructure, heat treatment will also be useful. The comparison of percentage elongation of the base alloy Al2014 and Al2014 reinforced with 15 wt.% Al_2_O_3_ particles before and after heat treatment is presented in Figure 5c. Figure 5c depicts the changes in the percentage elongation of the heat-treated Al2014 alloy and Al2014-15 wt.% Al_2_O_3_ MMC as contrasted to the base alloy and Al2014-15 wt.% Al_2_O_3_ MMC before heat treatment, respectively. It is observed that the produced composite before (1.56 ± 0.15%) and after T6 treatment (1.06 ± 0.11%) have better ductility than the base Al2014 matrix alloy before (11.21 ± 0.23%) and after heat treatment (6.26 ± 0.17%), respectively. A decrease in the percentage elongation of about 44.15% is observed for heat-treated Al2014 alloy when contrasted with as-cast Al2014 alloy before heat treatment. However, a reduction in the percentage elongation of about 32.05% is observed for heat-treated Al2014-15 wt.% Al_2_O_3_ composite, as contrasted to the composite produced before heat treatment.

It is observed that after heat treatment, there is a subsequent decrease in the percentage elongation for the composites with heat treatment, as compared to the composites produced without heat treatment. This is due to the fact that during the heat treatment, there may be a possibility for the formation of brittle intermetallic phases, which in turn dictates the ductility of the alloy matrix and the produced composites. The existence of inherently delicate phases and the presence of secondary or intermetallic phases are the potential locations for crack nucleation, which results in a decrease in the percentage elongation during static loading. The obtained percentage elongation results are in line with the results presented in the literature [46,47,48].

### 3.4. Tensile Fracture Behaviour

**Figure 6:**
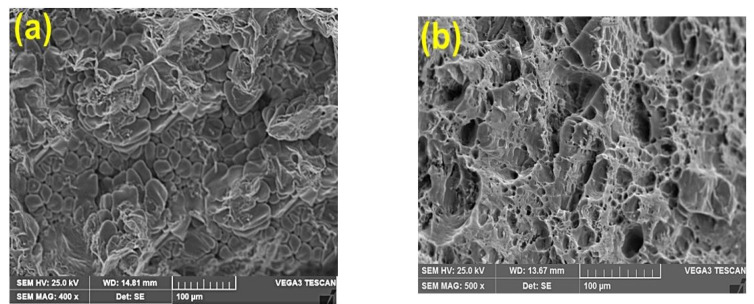

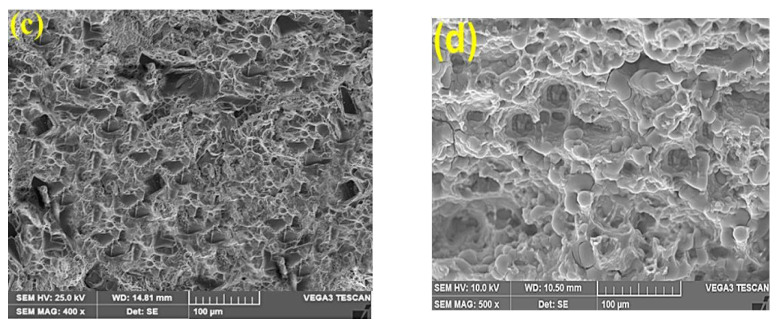
(**a**–**d**). (**a**) Fracto-graphic image of as-cast Al2014 before heat treatment, (**b**) Fracto-graphic image of as-cast Al2014 alloy after T6 heat treatment, (**c**) Al2014-15 wt.%, Al_2_O_3_ composite before heat treatment and (**d**) after heat treatment condition, respectively.

Figure 6a–d illustrates the fractured surfaces of both as-cast Al2014 alloy and Al2014-15 wt.% Al_2_O_3_ composite before and after the heat treatment conditions. The tensile fracture studies aim to understand the impact of heat treatment on the tensile fracture behavior of composites reinforced with maximum weight fractions.

Figure 6 shows the SEM photographs of the fractured surface of the Al2014 alloy and Al2014-15 wt.% Al_2_O_3_ composite before and after heat treatment. From Figure 6a, it is observed that the matrix alloy has larger dimples with voids; after being subjected to heat treatment, the matrix alloy tends to show a reduction in dimple size and voids, as shown in Figure 6b. The examined heat-treated composite sample in Figure 6d shows the dimples that are comparatively smaller in size with a rough structure when viewed on a microscopic scale, as compared to the composite produced without heat treatment Figure 6c. Microscopic voids intermingled with tear ridges surrounding the reinforcement and pockets of shallow dimples are also observed. This describes the ductile nature of the material systems studied due to the heat-treatment effect when compared with the composites produced without heat treatment.

### 3.5. Fatigue Test

In the present study, Al2014-15 wt.% Al_2_O_3_ composites are subjected to fatigue studies at T6 conditions, along with Al2014-15 wt.% Al_2_O_3_ composites and as-cast Al2014 matrix alloys at room temperature to understand the possible enhancement in the fatigue strength of the produced composites both for the T6 treated and as-cast samples at room temperature conditions.

**Figure 7:**
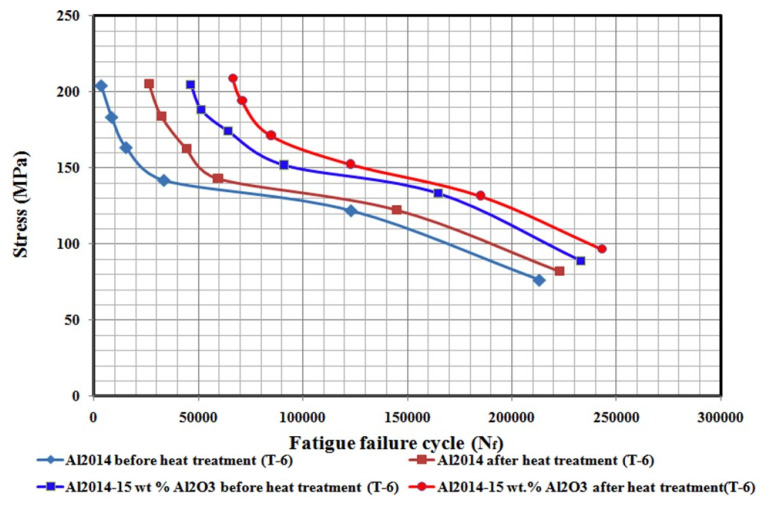
S–Ncurve for the comparison of fatigue behavior of Al2014 and Al2014-15 wt.% Al_2_O_3_ composites before and after heat treatment T6.

Figure 7 depicts the *S-N_f_* diagrams for the as-cast Al2014 alloy and Al2014-15 wt.% Al_2_O_3_ composites produced at as-cast and heat-treated conditions. From the figure, it is observed that the fatigue strength of the Al2014-15 wt.% Al_2_O_3_ composite produced, both for the as-cast and T6 heat-treated samples is higher than the as-cast Al2014 matrix alloy. Contrasted to the non-reinforced alloy, the maximum wt.% of the Al_2_O_3_ particles reported a significant enhancement in fatigue strength. This is because of the existence of hard Al_2_O_3_ particles and the substantial transfer of load to the reinforcement of the stiffer particles and the overall reduction in the total strain at given fatigue stress.

Consequently, MMCs reinforced with particles typically have fatigue durability limits and life spans higher than that of the non-reinforced metals. The positions of strains at persistent bands of slip in metals are commonly known to promote crack nucleation between the slip bands and grain boundaries and due to this, metal fatigue failure occurs. For MMCs, decreasing the particle size leads to a reduction in inter-particle separation at the respective volume fraction of the reinforcement. Fine particles serve as barriers to dislocation and refine the matrix slip length, which contributes to further obstacles to reversible slip movement or a decrease in stress position through the cycle slip refining [49].

By decreasing the reinforcing particle size, reinforcement fractures occur, which leads to premature fatigue life. The improvement in the fatigue strength can also be due to the reduced plastic and elastic strains induced by the modulus and work hardening rate, which both increase with the increased fraction volume of the reinforcement [50]. In addition, as noted by Murphy and Clyne [51], the reduction in porosity content can be concluded to enhance fatigue life. Furthermore, the strong interface relationship with the matrix and reinforcing particles is the key factor affecting the enhanced composites’ fatigue resistance.

Furthermore, the produced composites’ fatigue strength considerably improves at lower levels of stress than at high levels of stress. Thus, mean stress affects the fatigue response of the composites significantly. The high cycle fatigue behaviors of the 2xxx series Al alloy reinforced example situ SiC particles with various loading ratios are studied by Bonnen et al. [52]. It has been stated that fatigue life is decreased by an increase in mean stress. This is also a common activity in unreinforced metals. It is noticed that the fatigue life of both the matrix alloy and the composite produced is also reduced when the level of stress is increased.

However, the composite manufactured under T6 conditions displayed a drastically improved fatigue strength contrasted to the composite produced, in addition to the unreinforced matrix alloy, in all cases. This may relate to the lower porosity and improved mechanical properties. Clusters of reinforced particles, as well as defects such as porosity in cast composites, were reported to be stress raisers for the composites and to reduce their fatigue resistance [53]. The dispersion of the reinforced particles is much more uniform than the cast particles, as shown in the T6 composite micrographs. Uniform dispersion of reinforced particles decreases localized stress levels and improves fatigue life [54].

### 3.6. Wear Studies

#### 3.6.1. Impact of Variable Loads

**Figure 8:**
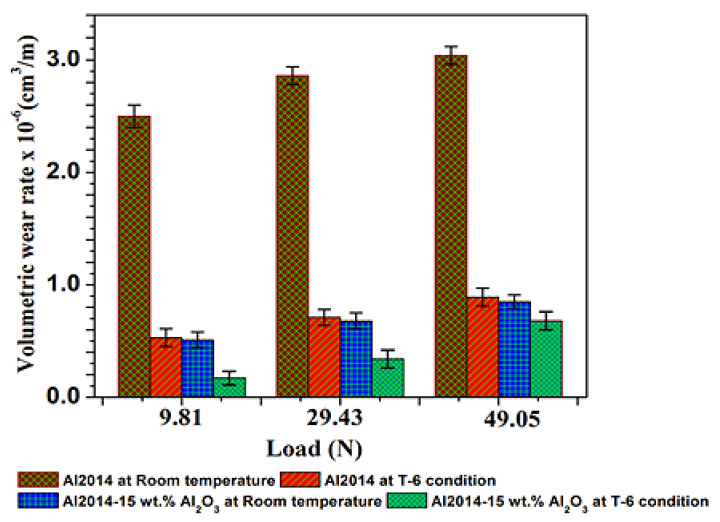
Comparison of the impact of the applied loads on the volumetric wear rate of Al2014 and Al2014-15 wt.% Al_2_O_3_ as-cast, T6 condition, and error bar represents standard deviation.

Figure 8 depicts the comparison of the before and after heat-treated Al2014 and Al2014-15 wt.% Al_2_O_3_ composite volumetric wear rate, which is investigated at three distinct loads (9.81, 29.43, and 49.05 N) by keeping the speed (400 rpm) and sliding distance (2000 m) constant. For every individual composite, three trials are conducted and the average values (w.r.t standard deviation) are presented in Table 4.

The difference in wear rate of the Al2014 matrix alloy and Al2014-15 wt.% Al_2_O_3_ composites before and after heat-treated conditions with variable loads is shown in Figure 8. As illustrated in Figure 8, it is observed that the Al2014 matrix alloy and Al2014-15 wt.% Al_2_O_3_ composites wear rate increases progressively with increments in the load up to 49.05 N before and after heat treatment. The maximum wear rate is observed in the un-heat treated Al2014 matrix alloy. The possible reason for this is extensive subsurface deformation, high adhesive metal-metal contact that assisted surface shear strain, and the absence of load-bearing particles [55,56,57].

Furthermore, the Al2014-15 wt.% Al_2_O_3_ composite before being subjected to heat treatment led to a lower wear rate because of the existence of ceramic Al_2_O_3_ particles, which resist the abrasion action and reduce the contact with the counter surface and soft matrix and contribute to some load, thereby reducing the wear rate [58]. However, under all the tested loads, the Al2014-15 wt.% Al_2_O_3_ composite under T6 conditions led to a decrease in the wear rate when contrasted with the heat-treated Al2014 matrix alloy and this is probably because of the formation of intermetallic precipitates and the matrix grain refinement and particulate addition, which also facilitate in improving the wear rate during the heat-treatment process. A similar trend is observed by other researchers [59].

#### 3.6.2. Impact of Variable Speed

**Figure 9:**
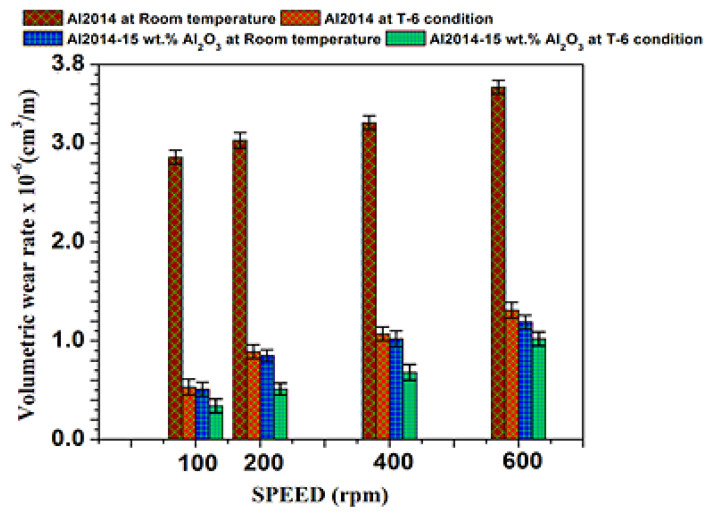
Impact of variable sliding speed on the volumetric wear rate of Al2014 and Al2014-15 wt.% Al_2_O_3_ at as-cast, T6 conditions and error bar represents standard deviation.

Figure 9 depicts the comparison of the un-heat treated and heat treated Al2014 and Al2014-15 wt.% Al_2_O_3_ composite volumetric wear rate, which is investigated at variable speeds (100, 200, 400, and 600 rpm) by keeping the load (49.05 N) and sliding distance (2000 m) constant. For every individual composite, three trials are conducted and the average values concerning standard deviation are presented in Table 5.

From Figure 9, it is observed that an increment in sliding speed leads to an increased wear rate of Al2014 and Al2014-15 wt.% Al_2_O_3_ composite before and after heat treatment. The advancement in wear rate with a rise in sliding speed may primarily be due to the greater temperature of the surface. As the sliding speed rises, the temperature of the surface increases, which facilitates the surface softening, leading to further damage to the surface and subsurface damage, which leads to the maximum wear rate. However, at all sliding speeds considered, the heat-treated Al2014-15 wt.% Al_2_O_3_ composite possesses lower wear rates when contrasted with the heat-treated Al2014 matrix alloy and un-heat treated Al2014 matrix alloy and Al2014-15 wt.% Al_2_O_3_ composite, respectively. The possible cause is the development of fine intermetallic particles that are uniformly aligned to strengthen the Al2014 matrix and protect the weaker matrix. Meanwhile, fine intermetallic particles (CuAl_2_), by providing more crystal nuclei during the solidification process, are shown to be very effective in the refinement of the grains.

#### 3.6.3. Impact of Variable Sliding Distance

The comparison of the un-heat treated and heat treated Al2014 and Al2014-15 wt.% Al_2_O_3_ composite volumetric wear rate is investigated at a variable sliding distance by keeping the load (49.05 N) and sliding speed (400 rpm) constant. For every test sample, three trials are conducted and the average values with standard deviation are presented in Table 6.

**Figure 10:**
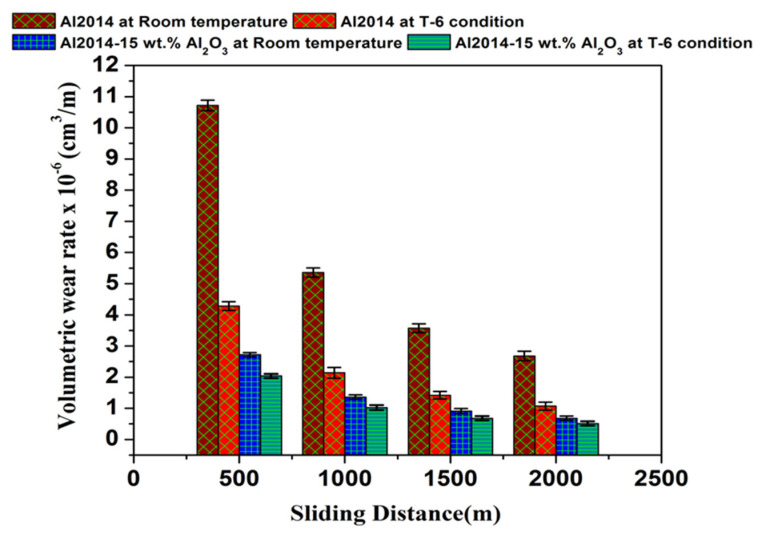
Impact of variable sliding distance on the volumetric wear rate of Al2014 and Al2014-15 wt.% Al_2_O_3_ at both as-cast, T6 conditions, and error bar represents standard deviation.

From Figure 10, it is observed that rising the sliding distance, leads to a reduction in the wear rate of all the material systems considered in the current study. Further reductions in the wear rate are observed for the as-cast Al2014 matrix alloy, as compared to heat-treated as-cast Al2014 matrix alloy and its Al2014-15 wt.% Al_2_O_3_ MMC with and without heat treatment. The rise in the rate of wear with the rise in the distance of sliding is probably because of (i) more intense contact time between the specimen contact surface and the rotating disc and (ii) the temperature at the disc interface increases with a rise in the sliding distance, so that the material is softened and appears to become plastic. Many other studies have reported similar results [60]. Furthermore, the existence of hard ceramic Al_2_O_3_ particles and uniform dissemination of the particles will act as a load-bearing element in the Al2014-15 wt.% Al_2_O_3_ MMC, which leads to a reduction in wear rate as the sliding distance increases. It is also observed that under T6 heat-treated conditions, the wear rate of the Al2014-15 wt.% Al_2_O_3_ composite decreases as contrasted to heat-treated Al2014 matrix alloy. However, the heat-treated Al2014-15 wt.% Al_2_O_3_ composite demonstrated a decrease in the wear rate in comparison to the matrix alloy. The decrease in the wear rate of the heat-treated composites may be due to the composites’ heavy plastic flow activity. Nevertheless, the heat-treated composite has lower wear rates at all sliding distances examined, as contrasted to the Al2014-15 wt.% Al_2_O_3_ MMC and Al2014 matrix alloy before heat treatment, respectively. Usually, the variation in the wear rate of the Al2014-15 wt.% Al_2_O_3_ MMC and as-cast alloy are considered at a higher sliding distance. Hence, at a higher sliding distance of 2000 m, a maximum of 60.07% reduction in wear rate was observed in the heat-treated Al2014 matrix alloy in comparison to the un-heat treated Al2014 matrix alloy and a 25% reduction in the wear rate was observed in the heat-treated Al2014-15 wt.% Al_2_O_3_ composite when contrasted to the un-heat treated Al2014-15 wt.% Al_2_O_3_ composite, respectively.

### 3.7. Morphological Worn Surface and Wear Debris Characteristics

The experimental outcomes of the impact of higher loads, sliding speed, and distance on the Al2014 matrix alloy and Al2014-15 wt.% Al_2_O_3_ composites, with and without heat treatment, reveal that Al2014-15 wt.% Al_2_O_3_ composites with heat treatment reinforced with fine particles (20 µm) are more effective and lead to better wear resistance, as compared to the un-heat treated Al2014-15 wt.% Al_2_O_3_ composite, as well as the unreinforced Al2014 alloy. Hence, in this section, the worn surface and debris morphological features of the Al2014 matrix alloy and Al2014-15 wt.% Al_2_O_3_ composites with and without heat treatment are depicted to understand the possible wear mechanism.

**Figure 11:**
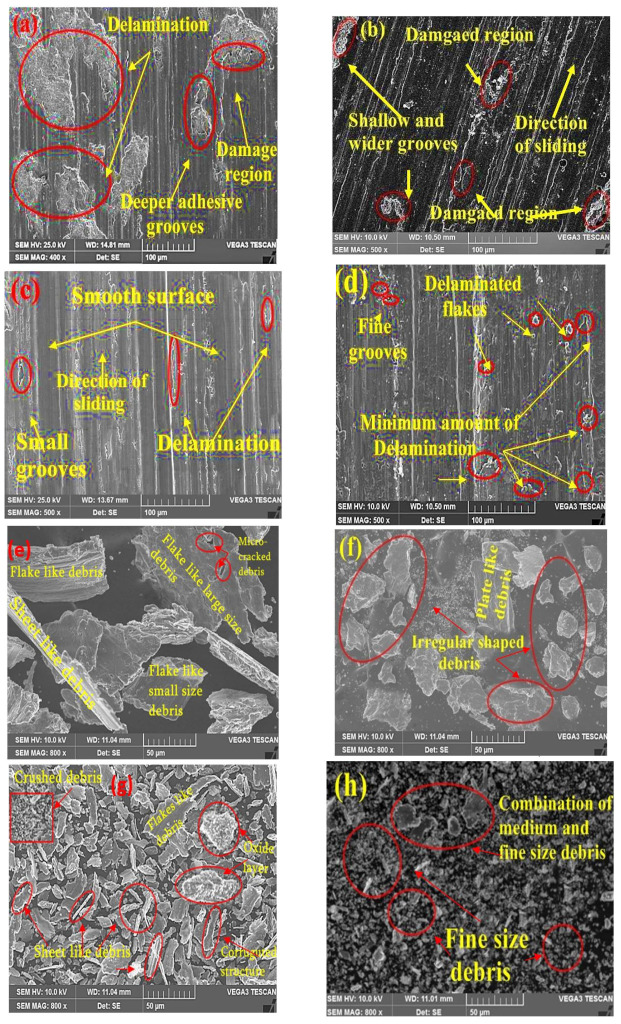
(**a**–**h**). (**a**–**d**) Worn surface electron microscopic images of (**a**) un-heat treated Al2014 matrix alloy, (**b**) heat-treated Al2014 matrix alloy, (**c**) un-heat treated Al2014-15 wt.% Al_2_O_3_ and (**d**) heat-treated Al2014-15 wt.% Al_2_O_3_ composites, respectively, with a load of 49.05 N, sliding speed of 400 rpm, and sliding distance of 2000 m. (**e**–**h**) Debris analysis of electron microscopic images of (**e**) Al2014 matrix alloy without heat treatment, (**f**) Al2014-15 wt.% Al_2_O_3_ composite with heat-treatment, (**g**) Al2014-15 wt.% Al_2_O_3_ composite without heat treatment and (**h**) Al2014-15 wt.% Al_2_O_3_ composites, respectively, at a load of 49.05 N, sliding speed of 400 rpm, and 2000 m.

Figure 11a–h depict the electron microscopy of wear tracks and debris of the heat-treated and un-heat treated Al2014 matrix alloy and Al2014-15 wt.% Al_2_O_3_ composites reinforced with fine size Al_2_O_3_ particles, which were examined at a load of 49.05 N, sliding speed of 400 rpm and sliding distance of 2000 m, respectively. The standard features observed for the unreinforced Al2014 alloy and all composites are the appearance of continuous and deep ploughing grooves; adhesion and abrasion phenomenons are observed parallel to the sliding direction.

Examination of the worn surfaces shows that both un-heat-treated Al2013 matrix alloy (Figure 11a) and heat-treated Al2014 matrix alloy (Figure 11b) are prevalent in adhesion wear mechanisms, whereas abrasion and delamination are prevalent wear mechanisms in the case of the composite produced with and without heat treatment (Figure 11c,d). It can be proposed that Al responds to an adhesive layer on the contacting surface with the iron in the EN31 steel disk because of the high-temperature friction produced during the sliding contact at the interface. The improved load transport capability and abrasion resistance of the composite were improved with the increase in wt% of the reinforcement particles. Figure 11c,d shows the worn surface images, which support the argument regarding the role of hard Al_2_O_3_ particles in improving the wear resistance of the composites for both heat-treated and untreated surfaces. The heat-treated Al2014 alloy in Figure 11b shows wider and shallow grooves on the surface, forming as the abrasive particles stick across the surface, and then removing or pushing the material in ridges along the sides of the grooves. Thus, fine grooves are observed when Al2014-15 wt.% Al_2_O_3_ composites were heat-treated with a relatively lower groove depth, as shown in Figure 11d, due to their higher hardness as compared to that of the heat-treated Al2014 matrix alloy. However, the extent of plastic deformation in the heat-treated composites appears to be less when contrasted to the composites produced before heat treatment. In contrast to the heat-treated and untreated matrix alloy, this finding supports the lower wear rate for the heat-treated composites.

Figure 11e indicates that the size of the sheet- and flake-like debris becomes much larger; indicating that the matrix becomes smooth with the above-mentioned wear testing conditions and results in the change from moderate to extreme wear. The micro-cracks were formed because of the occurrence of repeated stress while sliding at a greater load [61]. This means that the matrix is softer with a greater load of 49.05 N, sliding speed of 400 rpm and 2000 m, resulting in the change from mild to extreme wear. This wear debris suggests that the adhesive wear predominates along the direction of the sliding. Because of the adhesive aspect, the metal is sliced out in the form of the sheet- and flake-like debris at a higher load, speed, and sliding distance.

The study of the heat-treated debris from the Al2014 matrix alloy showed broad irregular profiles and unequal dimensions, as shown in Figure 11f. The formation of this kind of debris can be ascribed to an abrasive micro-cutting effect. Figure 11g demonstrates the wear debris in the form of a wavy pattern structure that occurs from the existence of fine-sized particles in the matrix, and it shows the existence of plate/flake/sheet-like and round shape oxides of Al_2_O_3_ debris. The oxide layers (Al_2_O_3_/Fe_2_O_3_) greatly decrease the composite wear rate under the wear conditions.

Even so, the heat-treated Al2014-15 wt.% Al_2_O_3_ composite, as shown in Figure 11h, wear debris becomes smaller and this decrease in debris size originates mainly from the lower probabilities of direct contact with the two surfaces used, which decreases the intensity of the micro-cutting effects and increased composite hardness when contrasted to the heat-treated Al2014 alloy.

**Figure 12:**
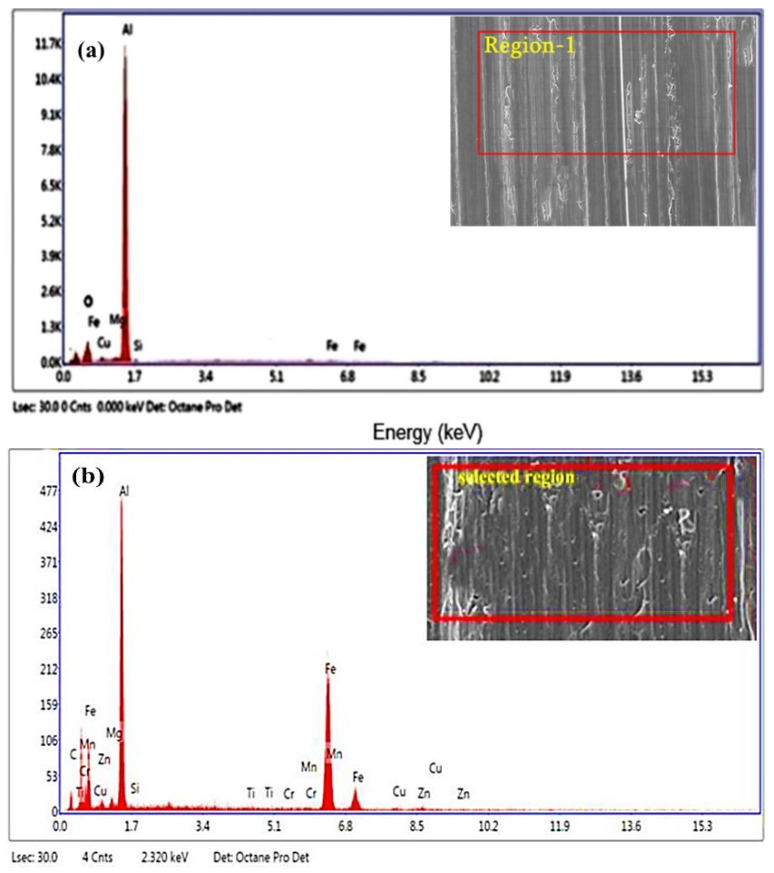
(**a**,**b**) Energy dispersive spectroscopy analysis of the worn surface of untreated and heat-treated Al2014-15 wt.% fine-sized alumina composite at 49.05 N, 600 rpm and 2000 m.

Figure 12a,b demonstrates the EDS spot of the untreated and heat-treated Al2014-15 wt.% Al_2_O_3_ composite. The study results show the presence of elements such as Cu, Mg, Si, Zn, Cr, and Fe. Furthermore, it is noticed that the extent of iron on the worn surfaces is at its maximum on the heat-treated composite sample (Figure 12b), as contrasted to the untreated composite Figure 12a, which results in the lower wear rate of the heat-treated composites. Another possible reason for the reduction in wear rate is because, upon heat treatment, there is an enhancement in the hardness of the heat-treated composites and the presence of more intermetallic phases, when compared with the untreated composite and matrix alloy, respectively.

## 4. Conclusions

- The SEM microphotographs of the Al2014 and Al2014-15 wt.% Al_2_O_3_ composites were successfully produced by the stir casting method with the two-step addition of Al_2_O_3_, which leads to the uniform dissemination of Al_2_O_3_ in the Al2014 matrix alloy.
- The microstructure of the heat-treated Al2014-15 wt.% Al_2_O_3_ composite is fragmented and thinner than the heat-treated Al2014 matrix alloy at 175 °C after aging for 10 h at a temperature of 175 °C, with Al_2_Cu precipitation.
- The SEM, XRD and EDAX studies confirm the presence of Al_2_Cu_1_, Al_2_Cu_1_Mg_1_ Al_2_Mg_1_O_4_ secondary intermetallic phases and reinforced Al_2_O_3_ particles in the heat-treated Al2014-15 wt.% Al_2_O_3_ composite and Al2014 matrix alloy, respectively.
- The microhardness of the heat-treated Al2014 matrix alloy and Al2014-15 wt.% Al_2_O_3_ composite is further enhanced by 10.36% and 32.71%, as contrasted to the untreated Al2014 matrix alloy and untreated Al2014-15 wt.% Al_2_O_3_ composite, respectively.
- An improvement in UTS of about 167.90 MPa and 326.39 MPa is observed in the heat-treated base alloy and Al2014-15 wt.% Al_2_O_3_ composite, respectively, as contrasted to the base alloy and Al2014-15 wt.% Al_2_O_3_ composite before heat treatment and an improvement in the YS of about 152.89 MPa and 263.23 MPa is observed in the heat-treated base alloy and Al2014-15 wt.% Al_2_O_3_ composite, respectively, as contrasted to the base alloy and Al2014-15 wt.% Al_2_O_3_ composite before heat treatment.
- Heat-treated Al2014-15 wt.% Al_2_O_3_ composites have shown lower ductility of about 1.06%, compared to untreated Al2014-15 wt.% Al_2_O_3_composites of about 1.56% and 6.26% in heat-treated Al2014 matrix alloys, as contrasted to unreinforced Al2014 matrix alloys of about 11.21% before heat treatment, respectively.
- The examined fractured surfaces of the heat-treated composite sample Al2014-15 wt.% Al_2_O_3_ show dimples that are comparatively smaller in size with rough structures when viewed on a microscopic scale, as compared to the Al2014-15 wt.% Al_2_O_3_ composite without heat treatment. Microscopic voids intermingled with tear ridges surrounding the reinforcement and pockets of shallow dimples are also observed. This describes the ductile nature of material systems studied, due to the heat treatment effect when compared with the composite produced without heat treatment.
- The fatigue strength of the composite increases after the addition of Al_2_O_3_ particles in Al2014 matrix alloys for both heat-treated and untreated conditions. The heat-treated Al2014-15 wt.% Al_2_O_3_ composite showed a noticeable improvement in fatigue strength, as contrasted to the untreated Al2014-15 wt.% Al_2_O_3_ compositeand Al2014 alloy.
- The wear rate of the heat-treated Al2014-15 wt.% of Al_2_O_3_ MMCis lower when contrasted to the untreated Al2014-15 wt.% of Al_2_O_3_ MMC, in addition to the untreated Al2014 matrix alloy, in all tested conditions, i.e., by varying the sliding speed, distance and applied load.
- The worn surface of the heat-treated Al2014-15 wt.% Al_2_O_3_ composites shows that the dominant wear mechanism is slightly plowing and contributes to improving the resistivity of delamination of the composite, as contrasted to the untreated Al2014-15 wt.% Al_2_O_3_ composite.

## Figures and Tables

**Table 1:** Chemical composition of Al2014 alloy by wt.%’ age.

Chemical Compositions	Si	Cu	Mn	Fe	Cr	Zn	Mg	Ti	Al
Al2014	0.7	4.5	0.83	0.2	0.01	0.19	0.63	0.06	Bal

**Table 2:** The base alloy matrix and particle reinforcement materials’ properties.

Material	Density (g/cc)	Elastic Modulus (Gpa)	Poisson’s Ratio	Hardness (HB500)	Tensile Strength (T)/Compressive Strength (C) (Mpa)
Al2014	2.8	72	2.7	130 BHN	410 (T)
Al_2_O_3_	3.69	300	0.21	1175	2100(C)

**Table 3:** Composition of Al2014 and Al2014-Al_2_O_3_ composites in wt.% by EDS analysis at heat-treated condition.

Elements	Al2014 (T6 Condition)	15 wt.% Alumina (T6 Condition)
O	0.24	20.27
Mg	0.87	2.16
Si	0.92	1.08
Fe	0.45	0.52
Cu	3.37	4.31
Ti	0.38	0.34
Cr	0.39	0.47
Zn	0.30	0.51
Mn	0.71	0.47
Al	92.37	69.87

**Table 4:** Volumetric wear rate values of before and after heat-treated Al2014 alloy and its Al2014-15 wt.% Al_2_O_3_ composite, with variable loads at constant speed and sliding distances.

Compositions of Composite Samples	Condition	Speed (rpm)	Sliding Distance (m)	Variable Load (N)		
				9.81	29.43	49.05
				Volumetric Wear Rate ∗ 10^−6^ (cm^3^/m)		
Al2014	RT	400	2000	2.50 ± 0.10	2.86 ± 0.08	3.04 ± 0.08
Al2014	T6	400	2000	0.53 ± 0.08	0.71 ± 0.07	0.89 ± 0.08
Al2014-15 wt.% Al_2_O_3_	RT	400	2000	0.51 ± 0.07	0.68 ± 0.07	0.85 ± 0.06
Al2014-15 wt.% Al_2_O_3_	T6	400	2000	0.17 ± 0.06	0.34 ± 0.08	0.68 ± 0.08

±—SD (standard deviation), RT—room temperature, T6—heat treated.

**Table 5:** Volumetric wear rate values of before and after heat-treated Al2014 and Al2014-15 wt.% Al_2_O_3_ composite with the variable speed at constant load and sliding distance.

Compositions of Composite Samples	Condition	Load (N)	Sliding Distance (m)	Variable Speed (rpm)	
				100	200
				Volumetric Wear Rate ∗ 10^−6^ (cm^3^/m)	
Al2014	RT	49.05	2000	2.86 ± 0.07	3.03 ± 0.08
Al2014	T-6	49.05	2000	0.53 ± 0.08	0.89 ± 0.07
Al2014-15 wt.% Al_2_O_3_					0.85 ± 0.06
	RT	49.05	2000	0.51 ± 0.07	
Al2014-15 wt.% Al_2_O_3_					0.51 ± 0.06
	T-6	49.05	2000	0.34 ± 0.07	

±—SD (standard deviation), RT—room temperature, T6—heat treated.

**Table 6:** The volumetric wear rate of before and after heat-treated Al2014 and Al2014-15 wt.% Al_2_O_3_ composite with variable sliding distance at constant load and speed.

Composition sof Composite Samples	Condition	Load (N)	Speed (rpm)	Variable Sliding Distance (rpm)			
				500	1000	1500	2000
				Volumetric Wear Rate ∗ 10^−6^ (cm^3^/m)			
Al2014	RT	49.05	400	10.72 ± 0.17	5.36 ± 0.15	3.57 ± 0.14	2.68 ± 0.15
Al2014	T-6	49.05	400	4.28 ± 0.14	2.14 ± 0.17	1.42 ± 0.12	1.07 ± 0.13
Al2014-15 wt.% Al_2_O_3_	RT	49.05	400	2.72 ± 0.07	1.36 ± 0.07	0.91 ± 0.08	0.68 ± 0.07
Al2014-15 wt.% Al_2_O_3_	T-6	49.05	400	2.04 ± 0.07	1.02 ± 0.08	0.68 ± 0.07	0.51 ± 0.07

±—SD (standard deviation), RT—room temperature, T6—heat treated.

## Data Availability

Available upon request from the corresponding author.
